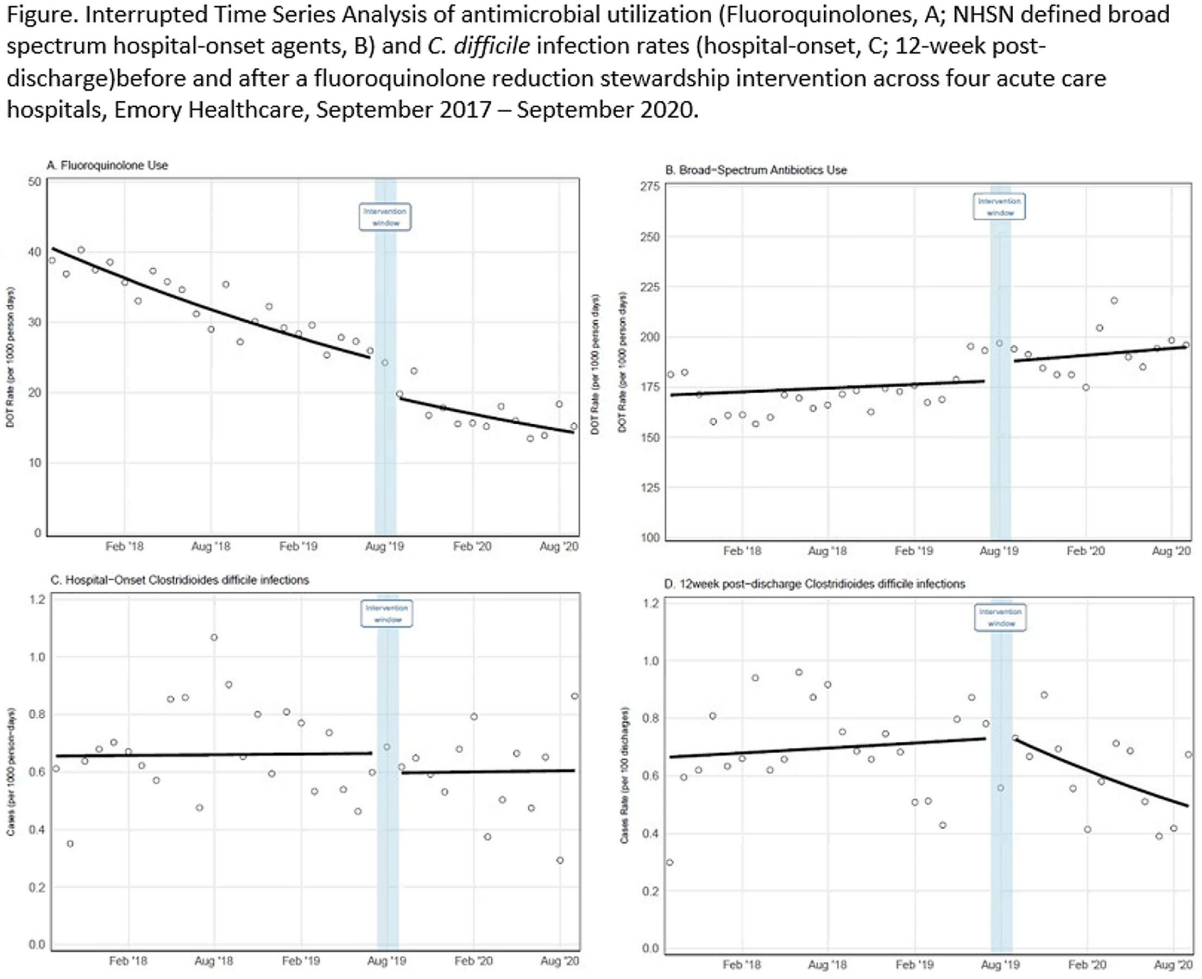# Reductions in Postdischarge *Clostridioides difficile* Infection after an Inpatient Health System Fluoroquinolone Stewardship

**DOI:** 10.1017/ash.2021.9

**Published:** 2021-07-29

**Authors:** K. Ashley Jones, Zanthia Wiley, Julianne Kubes, Mary Elizabeth Sexton, Benjamin Albrecht, Jesse Jacob, Jessica Howard-Anderson, Scott Fridkin, Udodirim Onwubiko

## Abstract

**Background:** Effective inpatient stewardship initiatives can improve antibiotic prescribing, but impact on outcomes like *Clostridioides difficile* infections (CDIs) is less apparent. However, the effect of inpatient stewardship efforts may extend to the postdischarge setting. We evaluated whether an intervention targeting inpatient fluoroquinolone (FQ) use in a large healthcare system reduced incidence of postdischarge CDI. **Methods:** In August 2019, 4 acute-care hospitals in a large healthcare system replaced standalone FQ orders with order sets containing decision support. Order sets redirected prescribers to syndrome order sets that prioritize alternative antibiotics. Monthly patient days (PDs) and antibiotic days of therapy (DOT) administered for FQs and NHSN-defined broad-spectrum hospital-onset (BS-HO) antibiotics were calculated using patient encounter data for the 23 months before and 13 months after the intervention (COVID-19 admissions in the previous 7 months). We evaluated hospital-onset CDI (HO-CDI) per 1,000 PD (defined as any positive test after hospital day 3) and 12-week postdischarge (PDC- CDI) per 100 discharges (any positive test within healthcare system <12 weeks after discharge). Interrupted time-series analysis using generalized estimating equation models with negative binomial link function was conducted; a sensitivity analysis with Medicare case-mix index (CMI) adjustment was also performed to control for differences after start of the COVID-19 pandemic. **Results:** Among 163,117 admissions, there were 683 HO-CDIs and 1,009 PDC-CDIs. Overall, FQ DOT per 1,000 PD decreased by 21% immediately after the intervention (level change; *P* < .05) and decreased at a consistent rate throughout the entire study period (−2% per month; *P* < .01) (Fig. 1). There was a nonsignificant 5% increase in BS-HO antibiotic use immediately after intervention and a continued increase in use after the intervention (0.3% per month; *P* = .37). HO-CDI rates were stable throughout the study period, with a nonsignificant level change decrease of 10% after the intervention. In contrast, there was a reversal in the trend in PDC-CDI rates from a 0.4% per month increase in the preintervention period to a 3% per month decrease in the postintervention period (*P* < .01). Sensitivity analysis with adjustment for facility-specific CMI produced similar results but with wider confidence intervals, as did an analysis with a distinct COVID-19 time point. **Conclusion:** Our systemwide intervention using order sets with decision support reduced inpatient FQ use by 21%. The intervention did not significantly reduce HO-CDI but significantly decreased the incidence of CDI within 12 weeks after discharge. Relying on outcome measures limited to inpatient setting may not reflect the full impact of inpatient stewardship efforts and incorporating postdischarge outcomes, such as CDI, should increasingly be considered.

**Funding:** No

**Disclosures:** None

**Figure 1.**